# Crystal Structure of *Crataeva tapia* Bark Protein (CrataBL) and Its Effect in Human Prostate Cancer Cell Lines

**DOI:** 10.1371/journal.pone.0064426

**Published:** 2013-06-18

**Authors:** Rodrigo da Silva Ferreira, Dongwen Zhou, Joana Gasperazzo Ferreira, Mariana Cristina Cabral Silva, Rosemeire Aparecida Silva-Lucca, Reinhard Mentele, Edgar Julian Paredes-Gamero, Thiago Carlos Bertolin, Maria Tereza dos Santos Correia, Patrícia Maria Guedes Paiva, Alla Gustchina, Alexander Wlodawer, Maria Luiza Vilela Oliva

**Affiliations:** 1 Departamento de Bioquímica, Universidade Federal de São Paulo, São Paulo, São Paulo, Brazil; 2 Macromolecular Crystallography Laboratory, Center for Cancer Research, National Cancer Institute, Frederick, Maryland, United States of America; 3 Centro de Engenharias e Ciências Exatas, Universidade Estadual do Oeste do Paraná, Toledo, Paraná, Brazil; 4 Institute of Clinical Neuroimmunology LMU, Max-Planck-Institute for Biochemistry, Martinsried, Munich, Germany; 5 Department for Protein Analytics, Max-Planck-Institute for Biochemistry, Martinsried, Munich, Germany; 6 Departamento de Biofísica, Universidade Federal de São Paulo, São Paulo, São Paulo, Brazil; 7 Departamento de Bioquímica, Universidade Federal de Pernambuco, Recife, Pernambuco, Brazil; Centro de Investigacion Principe Felipe and IBV-CSIC, Spain

## Abstract

A protein isolated from the bark of *Crataeva tapia* (CrataBL) is both a Kunitz-type plant protease inhibitor and a lectin. We have determined the amino acid sequence and three-dimensional structure of CrataBL, as well as characterized its selected biochemical and biological properties. We found two different isoforms of CrataBL isolated from the original source, differing in positions 31 (Pro/Leu); 92 (Ser/Leu); 93 (Ile/Thr); 95 (Arg/Gly) and 97 (Leu/Ser). CrataBL showed relatively weak inhibitory activity against trypsin (K_iapp_ = 43 µM) and was more potent against Factor Xa (K_iapp_ = 8.6 µM), but was not active against a number of other proteases. We have confirmed that CrataBL contains two glycosylation sites and forms a dimer at high concentration. The high-resolution crystal structures of two different crystal forms of isoform II verified the β-trefoil fold of CrataBL and have shown the presence of dimers consisting of two almost identical molecules making extensive contacts (∼645 Å^2^). The structure differs from those of the most closely related proteins by the lack of the N-terminal β-hairpin. In experiments aimed at investigating the biological properties of CrataBL, we have shown that addition of 40 µM of the protein for 48 h caused maximum growth inhibition in MTT assay (47% of DU145 cells and 43% of PC3 cells). The apoptosis of DU145 and PC3 cell lines was confirmed by flow cytometry using Annexin V/FITC and propidium iodide staining. Treatment with CrataBL resulted in the release of mitochondrial cytochrome *c* and in the activation of caspase-3 in DU145 and PC3 cells.

## Introduction

A large number of proteins are characterized by their β-trefoil fold [1]. Members of this superfamily share structural features but not necessarily other properties and their biological role may be widely different [2]. Prominent among them are plant protease inhibitors of the Kunitz type, called Kunitz-P or Kunitz-STI inhibitors [2], [3]. These proteins primarily inhibit serine proteases, although some also inhibit cysteine and aspartic proteases [4]. Kunitz-P inhibitors are characterized by molecular mass of about 20,000 Da (for the whole protein or a domain), low content of cysteine residues, and one or two reactive sites that are the basis of their inhibitory activity. However, some structurally related β-trefoil proteins are not protease inhibitors at all, but exhibit varied other properties, for example chlorophyll binding [5], taste modification (miraculin) [6], binding to cytokine receptors (IL-1β) [7], ribosome poisoning (ricin) [8] or carbohydrate binding, exemplified by the *Clitocybe nebularis* lectin, CNL [9].

Plant lectins are proteins that possess at least one non-catalytic domain that binds reversibly and specifically mono- or oligosaccharides [10], [11]. Owing to these properties, such proteins are being utilized in characterization of glycoconjugates and in cell-surface or cell-cell architecture studies [12], [13]. A few protease inhibitors also show lectin activity, an example provided by the inhibitor isolated from the epidermis of the eel, named Eel-CPI-1 [14]. Troncoso et al. [15] isolated an inhibitor with lectin properties from *Peltophorum dubium* seeds which decreases the viability of the Nb2 rat lymphoma cells.

*Crataeva tapia* (also known as *Crateva tapia*) belongs to the Capparaceae family that is found in northeastern Brazil. A protein from the *Crataeva tapia* bark has been purified using reversed micelles in isooctane [16] and through chromatographic processes [17]. Named CrataBL (*Crataeva tapia* Bark Lectin), this protein was shown to possess some specificity for binding glucose and galactose [17]. A number of its biological properties have been characterized, including delay in clot formation by impairing the intrinsic pathway of coagulation cascade [18]. In addition, CrataBL was shown to exhibit anti-inflammatory, analgesic [19], insecticidal [17], and anti-tumor [19] activities.

Prostate cancer is the most common cancer in men [20], with DU145 and PC3 being the most extensively studied prostate cancer cell lines that serve as *in vitro* models for cancer treatment studies [21], [22], [23]. Prostate cancer is associated with a high level of expression of proteases [24] and glycoproteins [25], making CrataBL a potentially useful tool for studies involving prostate cancer cell lines, due to its combination of properties that include both protease inhibition and carbohydrate binding.

In this work, we determined the amino acid sequence and the three-dimensional structure of CrataBL and also investigated a range of biochemical activities, comparing them to the properties of other members of this superfamily. Subsequently, we characterized the effect of CrataBL on growth and stability of human prostate cancer cell lines.

## Materials and Methods

### Isolation of CrataBL

Isolation of the protein was performed according to the procedure of Araújo et al. [17]. Briefly, 10% (w/v) extracts from *Crataeva tapia* bark were fractionated with 30–60% ammonium sulfate. The fraction containing the protein was dialyzed against 10 mM phosphate citrate buffer pH 5.5 and applied to a column of CM-cellulose equilibrated with the same buffer. Adsorbed protein was eluted with an equilibration buffer containing 0.5 M NaCl and contents of the single peak were submitted to size exclusion chromatography on Superdex 75 column, equilibrated in 0.15 M NaCl using an ΔKTA Purifier (GE Healthcare, Uppsala, Sweden). This was followed by high performance liquid chromatography (HPLC) on a C18 column, to confirm the homogeneity of the sample. The elutions were monitored at 280 nm.

### Protein concentration and carbohydrate assays

Protein determination was carried out as described by Lowry et al. [26] using bovine serum albumin (BSA) as the standard. The sugar content of CrataBL was determined by phenol-sulfuric acid method of Masuko et al. [27], with mannose at concentrations of 2, 4, 6, 8, and 10 µg as a standard.

### MALDI-TOF/MS analysis

The molecular mass of CrataBL was analyzed by MALDI-TOF/MS. 1 µL CrataBL (2 mg/mL) was added to 1 µL of α-cyano-4-hydroxycinnamic acid (10 mg/mL) matrix solution, spotted onto a stainless steel MALDI target plate and dried at room temperature before analysis. Mass spectra were obtained with a Bruker Daltonics Microflex LT instrument (Billerica, USA) operating in linear, positive ion mode, previously calibrated with insulin, ubiquitin, cytochrome C, and myoglobin. For the analysis of the protein, mass spectra were acquired using the following instrument parameters: pulsed ion extraction delay of 30 ns, ion source voltage one, 20 kV, ion source voltage two, 18.65 kV, and ion source lens voltage 7.1 kV. For each sample, mass spectra were acquired by accumulating 50 laser shots at 50% laser power in the m/z range of 8000–25000 Da.

### Primary structure of CrataBL

The sequencing of the native protein was performed by Edman degradation [28]. The sample was reduced, alkylated, and then submitted to digestion with trypsin, chymotrypsin, Asp-N, and Lys-C endopeptidases (sequencing grade, Roche, Germany). Fragments resulting from the treatment with endopeptidases were purified on a 1×150 mm Jupiter Proteo 90A column (Phenomenex, Aschaffenburg, Germany) at a flow rate of 0.1 mL/min, using an acetonitrile gradient (0–60%) with 0.1% (v/v) trifluoroacetic acid for 40 min. Sequencing was performed on an automatic gas-phase sequencer (492cLC; Applied Biosystems, Foster City, USA). The glycosylated peptides were analyzed by mass spectrometry to determine the molecular mass of the carbohydrate moieties. Sequence similarities were assessed with the program BLAST against the NCBI protein database [29]. The sequences were aligned using the program MULTALIN [30].

### Enzyme inhibition assays

The inhibitory activity of CrataBL against proteases was measured using specific chromogenic or fluorogenic substrates in 96-well microtiter plates at 37°C. The enzymes (human Factor Xa, bovine trypsin, bovine chymotrypsin, human plasma kallikrein (HuPK), porcine pancreatic kallikrein (PoPK), human neutrophil elastase (HNE), human plasmin, and papain) were pre-incubated with either CrataBL or with solvent for 10 min. The reactions were initiated by the addition of the substrates, and the color or fluorescence were monitored continuously at 405 nm using a spectrophotometer (Packard, SpectraCount), or at 380/460 nm using a Hitachi F-2000 spectrofluorimeter (Tokyo, Japan), respectively, for 30 min, and stopped with the addition of 50 µL of 30% (v/v) acetic acid. Enzymatic activities were analyzed in the following buffers (final concentrations): human factor Xa (21 nM in 0.02 M Tris-HCl, pH 7.4 containing 0.14 M NaCl, 5.0 mM CaCl_2_ and 0.1% bovine serum albumin; 0.57 mM Boc-Ile-Glu-Gly-Arg-AMC); bovine trypsin (40 nM in 0.1 M Tris-HCl, pH 8.0 containing 0.02% (v/v) CaCl_2_; 0.8 mM Bz-Arg-pNan); bovine chymotrypsin (88 nM in 0.05 M Tris-HCl, pH 8.0; 0.8 mM Suc-Phe-pNan); HuPK (14.7 nM in 0.1 M Tris-HCl, pH 8.0 containing 0.5 M NaCl; 0.4 mM H-D-Pro-Phe-Arg-pNan); PoPK (2.6 nM in 0.1 M Tris-HCl, pH 9.0; 0.16 mM HD-Val-Leu-Arg-pNan); HNE (1.3 nM in 0.05 M Tris-HCl, pH 7.0 containing 0.5 M NaCl; 0.88 mM MeO-Suc-Ala-Ala-Pro-Val-pNan); human plasmin (3.5 nM in 0.1 M Tris-HCl, pH 7.4 containing 0.2 M NaCl; 0.9 mM H-D-Val-Leu-Lys-pNan) and papain (87 nM in 0.1 M Na_2_HPO_4_, pH 6.3 containing 0.4 M NaCl, 0.01 M EDTA; 0.4 mM Z-Phe-Arg-pNan). The apparent K_iapp_ values were determined by fitting the experimental points to the equation for slow-tight binding [31] with the help of the Grafit program, version 4.0 (Erithacus Software, Staines, UK) [32].

### Reactive site determination by affinity chromatography on trypsin-Sepharose

A sample containing 1.8 mg of protein in 0.1 M Tris-HCl buffer pH 8.0 was applied to a 1.0 mL trypsin-Sepharose column equilibrated with the same buffer. The inhibitor was eluted by 0.5 M KCl/HCl, pH 2.0 and kept in this pH until the N-terminal determination step.

### Protein crystallization

A highly purified protein sample pooled from a Superdex 75 column was dialyzed into a buffer consisting of 20 mM Tris pH 8.0, 100 mM NaCl, and 3% glycerol, and then was concentrated to 8.5 mg/mL using Centrifugal Filter Units (Millipore, Billerica, MA). Initial crystallization trials were performed using a Phoenix robot (Art Robbins Instruments, Mountain View, CA). A number of hits were obtained from several different crystallization screens [JCSG (Qiagen, Valencia, CA), Crystal Screen HT, Index (Hampton Research, Aliso Viejo, CA), and Wizard1-2 (Emerald Biosystems, Bedford, MA)]. Diffraction quality crystals were obtained from screens JCSG-CORE-II D12 (0.2 M Li_2_SO_4_, 30% PEG3350) and G8 (0.16 M ammonium sulfate, 0.08 M sodium acetate pH 4.6, 20% PEG4000, 20% glycerol). Crystals appeared on the second day and grew to the full size within several days at 20°C.

### X-ray data collection and processing

Diffraction data were collected at the Southeast Regional Collaborative Access Team (SER-CAT) beamline 22-ID at the Advanced Photon Source, Argonne National Laboratory. Single crystals were transferred to a cryoprotectant solution (mother liquor with extra 10–20% glycerol) for approximately 2 min and then were flash frozen at 100 K in a stream of liquid nitrogen. A crystal from JCSG-CORE-II condition G8 (named form I) diffracted X-rays to the resolution of 1.5 Å. Diffraction data were indexed, integrated, and scaled with the program XDS [33]. The crystal belongs to the space group *C*2 with unit cell parameters a = 114.9 Å, b = 46.2 Å, c = 71.5 Å, β = 103.4° and contains a dimer of CrataBL in the asymmetric unit. The estimated Matthews coefficient is 2.26 Å^3^ Da^−1^, corresponding to 45.5% solvent content. A crystal from JCSG-CORE-II condition D12 (named form II) diffracted X-rays to the resolution of 1.75 Å. Diffraction data were also processed with the program XDS. The crystal also belongs to the space group *C*2 but with different unit cell parameters, a = 95.6 Å, b = 76.3 Å, c = 62.3 Å, β = 120.1°, with a single CrataBL dimer in the asymmetric unit. The estimated Matthews coefficient is 2.66 Å^3^ Da^−1^, corresponding to 53.9% solvent content. Data processing statistics for both crystal forms are shown in **Table 1**.

**Table 1:** Data collection and structure refinement.

Data collection		
	CrataBL-form I	CrataBL-form II
Space group	*C*2	*C*2
Molecules/a.u.	2	2
Unit cell *a*, *b*, *c* (Å);	114.9, 46.2, 71.5	95.6, 76.3, 62.3
β (°)	103.4	120.1
Resolution (Å)*	20.0-1.50 (1.75-1.50)	20.0-1.75 (1.85-1.75)
*R*_merge_ † (%)	6.5 (93.2)	5.6 (35.8)
No. of reflections (measured/unique)	427,881/58,600	257,578/38,040
<*I*/σ*I*>	15.72 (2.43)	20.21 (3.87)
Completeness (%)	99.9 (100.0)	97.0 (96.1)
Redundancy	7.3 (7.3)	6.8 (5.5)
**Refinement**		
Resolution (Å)	19.95-1.50	19.98-1.75
No. of reflections (refinement/*R*_free_)	57,427/1,172	37,089/951
*R*/*R*_free_ ‡	0.173/0.208	0.185/0.232
No. atoms		
Protein	2572	2576
Ligands	98	154
Water	394	297
R.m.s. deviations from ideality		
Bond lengths (Å)	0.014	0.017
Bond angles (°)	1.8	1.9
PDB ID	4IHZ	4II0

* The highest resolution shell is shown in parentheses.

† *R*_merge_ = Σ_h_Σ_i_|*I*_i_-〈*I*〉|/Σ_h_Σ_i_ *I*_i_, where I_i_ is the observed intensity of the i-th measurement of reflection h, and 〈I〉 is the average intensity of that reflection obtained from multiple observations.

‡ *R* = Σ||*F_o_*|-|*F_c_*||/Σ|*F_o_*|, where F_o_ and F_c_ are the observed and calculated structure factors, respectively, calculated for all data. *R*_free_ was defined in [37].

### Structure determination and refinement

The structure of CrataBL was initially determined using diffraction data of the crystal form I and the resulting coordinates were subsequently used as the starting model for molecular replacement with data collected for crystal form II. The structure of CrataBL was solved by molecular replacement with the program Phaser [34], [35] using the structure of water-soluble chlorophyll protein (PDB ID: 2DRE) as a starting model. The latter protein was chosen due to its highest primary structure identity with CrataBL (>30%). A unique solution representing two molecules in the asymmetric unit was found for data between 20.0 and 2.5 Å, with a Log-Likelihood gain of 122.3. The resulting electron density map was fully interpretable, verifying the correctness of the solution. Further refinement was performed with REFMAC5 [36] and PHENIX [34], using all data between 20 and 1.5 Å, after setting aside 2% of randomly selected reflections (1172 total) for calculation of R_free_ [37]. Isotropic individual temperature factors were refined, with the TLS parameters added in the final stages of refinement. After several further rounds of automated refinement and manual correction using COOT [38], the structural model was finally refined to an R-factor of 17.3% and R_free_ of 20.8%. The structure of crystal form II was solved with Phaser and the form I coordinates as the starting model; its refinement was carried out using a protocol similar to the one reported for crystal form I. The final model was refined to 1.75 Å resolution, resulting in an R-factor of 18.5% and R_free_ of 23.2%. The refinement statistics for the structures of CrataBL in the two crystal forms are shown in **Table 1**. The structures were compared using the Dali server [39] with the set of Protein Data Bank structures with less than 90% sequence identity.

### Cell viability assay

#### Cell Lines

The cell lines DU145 (HTB-81™) and PC3 (CRL-1435™) were purchased from ATCC®. Cells were maintained in the Roswell Park Memorial Institute (RPMI) media supplemented with 10% (v/v) heat-inactivated fetal bovine serum (FBS), 100 µg/mL of streptomycin and 100 IU/mL of penicillin, at 37°C in an atmosphere of 5% CO_2_. Cell viability was determined by the modified colorimetric assay utilizing 3-(4,5-dimethylthiazol-2-yl)-2,5-diphenyltetrazolium bromide (MTT) (Sigma Aldrich, St. Louis, USA). DU145 or PC3 were plated in 96-well plates (TPP, Trasadingen, Switzerland) at a density of 5.0×10^3^ cells per well for 24 h. Subsequently the cells were treated with different concentrations of CrataBL (5, 10, 20, and 40 µM) or soybean trypsin inhibitor (SbTI) (5, 25, 50, 100 µM) prepared in RPMI media at 37°C in an atmosphere of 5% CO_2_. After 24, 48, and 72 h of culture, 10 µL of MTT (5 mg/mL in phosphate buffered saline, PBS) were added to the wells (2 h, 37°C), followed by the removal of the medium and addition of 100 µL/well of dimethyl sulfoxide (DMSO) (Sigma Aldrich, St. Louis, USA) to solubilize the crystals of formazan. The absorbance was measured at 540 nm using a spectrophotometer (Packard, SpectraCount). Each experiment was performed in triplicate.

### Cell death analysis by flow cytometry

DU145 and PC3 cells (1×10^5^ cells) were seeded on 6-well plates (TPP, Trasadingen, Switzerland) for 24 h for complete adhesion. The wells were subsequently washed three times with RPMI media without FBS (at incubation periods of 15 min at 37°C and 5% CO_2_). The cells were incubated for 24 h in RPMI media without FBS to synchronize the cell cycle. Thereafter, the cells were treated with CrataBL (40 µM) for 24 and 48 h in RPMI without FBS at 37°C and 5% CO_2_. At the end of each incubation, the cells were removed from the plate using trypsin-EDTA solution (Cultilab, Campinas, Brazil) [40], transferred to cytometry tubes, washed with 500 µL of binding buffer (BD PharMingen kit), centrifuged, and resuspended in 50 µL of the same buffer also containing 3 µL of Annexin V-FITC and 5 µL of propidium iodide (PI). The reaction mixture was incubated for 30 min in the dark, and then 300 µL of the same binding buffer was added to each tube and analyzed by flow cytometry (FACSCalibur, BD, San Diego, USA) [41], [42].

### Analysis of mitochondrial cytochrome c release

DU145 and PC3 cells were seeded on 13-mm glass coverslips (5×10^4^ cells/well), placed in 24-well plates and incubated for 24 h at 37°C and 5% CO_2_ for full adherence. The cells were subsequently incubated in the RPMI media without FBS for 24 h, followed by the treatment with 40 µM CrataBL in the RPMI media without FBS, and incubated at 37°C and 5% CO_2_ for 12 h. At the end of the treatment, the cells were washed with PBS and the mitochondria were labeled with Mitotracker Deep Red 633 (1∶500) (Molecular Probes) for 20 min in the dark and fixed with 2% (v/v) paraformaldehyde in PBS for 15 min. The cells were washed again three times with PBS containing 0.01 M glycine and then permeabilized with 0.01% saponin for 15 min. The cells were subsequently stained for 2 h with the primary antibody, mouse anti-cytochrome c (1∶400) (R&D Systems), followed by staining for 40 min with the secondary antibody, mouse anti-IgG conjugated with Alexa Fluor 488 (1∶500) (Invitrogen, CA, USA). Cell nuclei were labeled with the fluorophore DAPI (1∶3000) (Invitrogen, CA, USA) for 30 min and then fixed to slides with 7 µL of fluoromount G (Electron Microscopy Sciences). Visualization and measurements of the fluorescence slides were performed in a confocal scanning microscope Carl Zeiss LSM780 laser model, 63× objective, using oil immersion and a numerical aperture of 1.43. The program used for image acquisition was Zen 2010.

### Caspase 3 activity by flow cytometry

DU145 and PC3 cells (1×10^5^ cells) were seeded in each well of a 6-well plate (TPP, Trasadingen, Switzerland) containing RPMI media supplemented with 10% FBS for adhesion. The cells were then washed three times with RPMI media without FBS and incubated for 24 h. Twenty-four hours later the medium was replaced with 40 µM of CrataBL and further incubated for 48 h at 37°C and 5% CO_2_. At the end of the treatment, the cells were removed from the plate using a trypsin-EDTA solution (Cultilab, Campinas, Brazil) [40], transferred to cytometry tubes and fixed with 100 µL of 2% (v/v) paraformaldehyde for 30 min at room temperature. The cells were resuspended after centrifugation in 200 µL of 0.01% glycine in PBS and incubated for 15 min at room temperature. The cells were then resuspended again in 200 µL of 0.01% saponin in PBS and incubated for 15 min at room temperature. Finally, the cells were incubated with 10 µL of cleaved caspase 3 AlexaFluor 488-conjugated antibody (BD, San Diego, USA) for 40 min. The results were analyzed by flow cytometry (FACSCalibur - BD, San Diego, USA).

### Statistical analysis

Differences between the mean values were analyzed using a one-way ANOVA followed by Tukey's test. Values were considered to be significant when *p<0.05; **p<0.01; ***p<0.001.

### Data deposition

The atomic coordinates and structure factors have been deposited with the Protein Data Bank, www.rcsb.org (PDB accession codes 4IHZ and 4II0 for crystal forms I and II, respectively). The protein sequence data reported here will appear in the Uniprot Knowledgebase under the accession numbers B8WI86 and C0HJA4.

## Results and Discussion

### Purification of CrataBL and assessment of its activities

**Figure 1:**
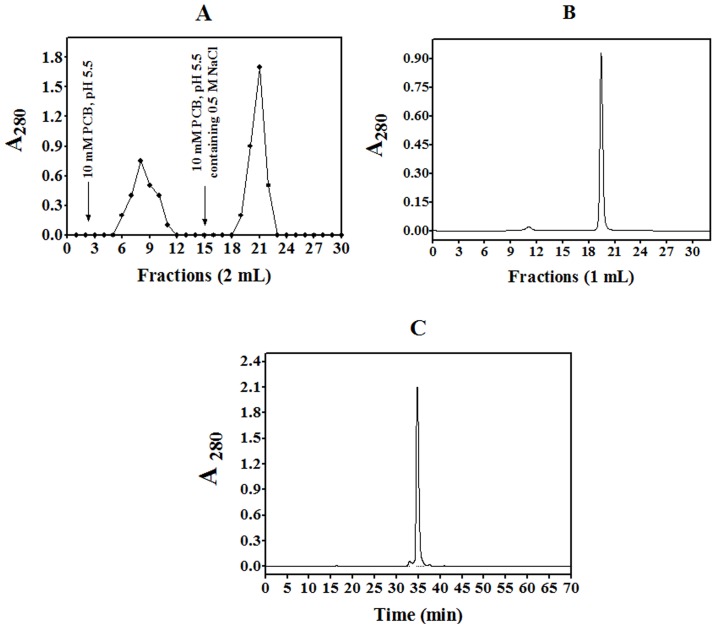
Purification of CrataBL. (**A**) The fraction 30–60% was submitted to ion-exchange chromatography on CM cellulose equilibrated with 10 mM phosphate citrate buffer (PCB), pH 5.5. Two mL fractions were collected at the flow rate 0.3 mL/min. An arrow indicates elution with 10 mM phosphate citrate buffer, pH 5.5, containing 0.5 M NaCl; (**B**) Size exclusion chromatography on Superdex 75 equilibrated with 0.15 M NaCl at the flow rate 0.5 mL/min. (**C**) The protein fraction was eluted with a linear gradient (5–100%) of 90% acetonitrile in 0.1% TFA in Milli-Q water (solvent B) at the flow rate of 0.7 mL/min (*t* = 0.1 min, 5% B; *t* = 5 min, 5% B; *t* = 30 min, 40% B; *t* = 50 min, 50% B; *t* = 60 min, 100% B; *t* = 65–68 min, 0% B).

CrataBL is a basic protein (pI>10) that could be purified in a relatively simple way [17]. The initial steps involved extraction in 0.15 M NaCl and ion exchange chromatography in CM-Cellulose (**Figure 1A**). Size exclusion chromatography on a Superdex 75 column (**Figure 1B**) allowed purification of the protein into a homogeneous form suitable for structural studies, with only a single major peak visible after reverse-phase chromatography on a C_18_ HPLC column (**Figure 1C**). Native protein migrates mostly as a homodimer at the concentration used in the crystallization trials (see below).

Lectin and hemagglutinating activities of CrataBL were noted previously [17] and these properties have not been analyzed further. However, we have assessed the ability of CrataBL to function as a protease inhibitor. We found that it could inhibit bovine trypsin and Factor Xa, but no other serine peptidases including chymotrypsin, plasmin, human neutrophil elastase, human plasma kallikrein, porcine pancreatic kallikrein, or a cysteine protease papain (**Table 2**). K_iapp_, calculated using the equation described by Morrison et al. [32], was 43 µM for trypsin and 8.6 µM for Factor Xa, indicating that CrataBL is a relatively weak inhibitor, much less potent than some other inhibitors that belong to the same family which are often nanomolar of even picomolar [43], [44].

**Table 2:** Inhibitory properties of CrataBL.

Enzyme	K_iapp_
Trypsin	43 µM
Human Factor Xa	8.6 µM
Human neutrophil elastase	NI
Human plasma kallikrein	NI
Papain	NI
Human plasmin	NI
Porcine pancreatic kallikrein	NI
Bovine chymotrypsin	NI

Enzyme inhibition activity was previously described; (NI) no inhibition detected.

### Primary structure, glycosylation, and biochemical properties of CrataBL

**Figure 2:**
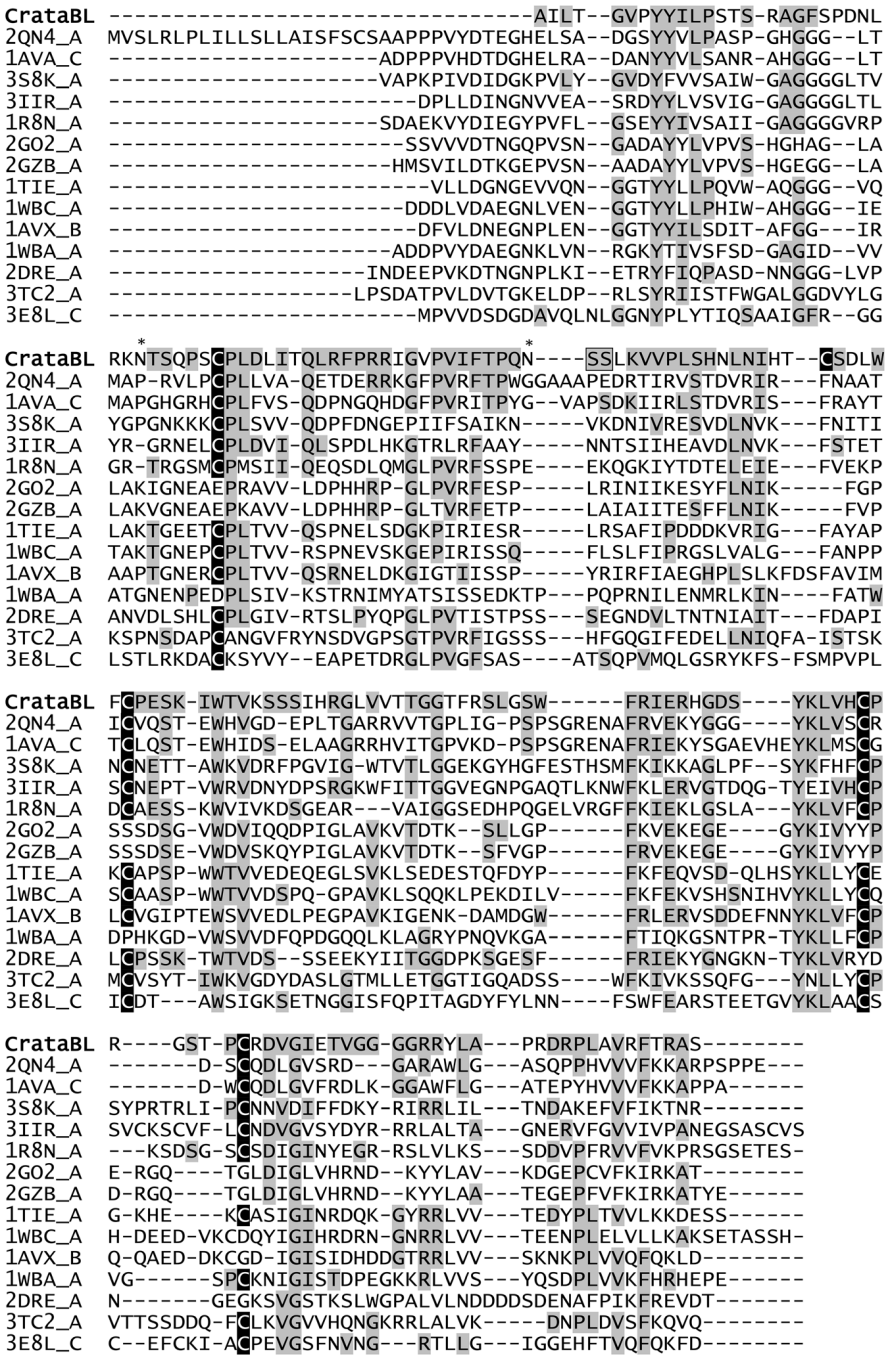
Similarity of CrataBL to others proteins. A comparison of the amino acid sequence of isoform I of CrataBL with the sequences of structurally similar proteins, as determined with the program Dali [39]. Cysteine residues are shown in black boxes and highly conserved amino acids are highlighted in gray. Residue positions P1 and P1′, between which the proteolytic cleavage occurs, are denoted in box. Asterisks indicate glycosylation sites in CrataBL. The sequence of isoform II differs in positions 31 (Pro/Leu); 92 (Ser/Leu); 93 (Ile/Thr); 95 (Arg/Gly) and 97 (Leu/Ser).

The primary structure of mature CrataBL consists of a single polypeptide 164 or 165 amino acid long, present in at least two distinct isoforms, differing in positions 31 (Pro/Leu); 92 (Ser/Leu); 93 (Ile/Thr); 95 (Arg/Gly) and 97 (Leu/Ser) (**Figure 2**). The C-terminal Gly165 may be present in only a fraction of the molecules. The presence of five cysteine residues indicated a possibility of forming two intramolecular disulfide bonds, the presence of which was verified by the crystal structures (see below).

**Figure 3:**
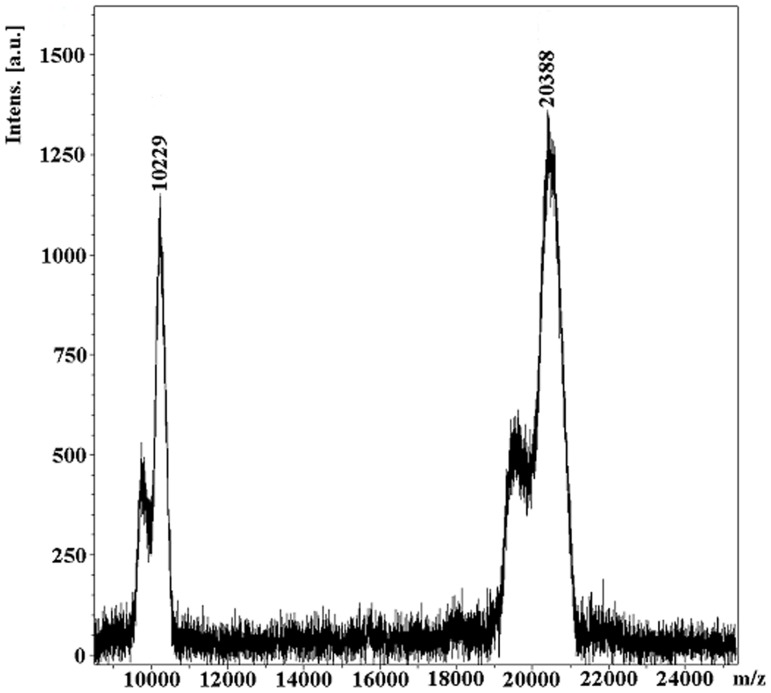
Molecular mass of glycosylated CrataBL determined by MALDI-TOF/MS.

Araújo et al. [17] estimated the molecular mass of CrataBL at about 21 KDa on SDS-PAGE and have shown by Schiff staining that the protein is glycosylated. Further analysis of glycosylation by mass spectrometry and protein sequence determination has shown the presence of N-linked oligosaccharides at Asn27 and Asn57. MALDI-TOF MS analysis of the molecular mass of native protein gave a peak at m/z 20388, with a shoulder at about 19700 (M+H)^+^, indicating the presence of an isoform. An additional peak of 10229 is due to molecular ion (M+2H)^+^ (**Figure 3**). The phenol-sulfuric acid method [27] was used to determine the concentration of carbohydrate on the protein, indicating ∼6% carbohydrate content.

The molecular mass of carbohydrate moieties were estimated by the differences between the mass spectrometry of the glycosylated peptides and their amino acid sequences. In the case of the Asn57, the molecular weight is shown to be 1,170 Da whereas at Asn27, additional signals of 162 Da or more were detected (data not shown).

The sequences of both isoforms of CrataBL show high similarity to a variety of proteins with β-trefoil fold (**Figure 2**), including Kunitz type inhibitors from the STI (soybean trypsin inhibitor) family [45]. Such inhibitors function by tightly binding to their target protease and inserting their reactive loop into its active site, sometimes also acting as slow substrates [2].

**Figure 4:**
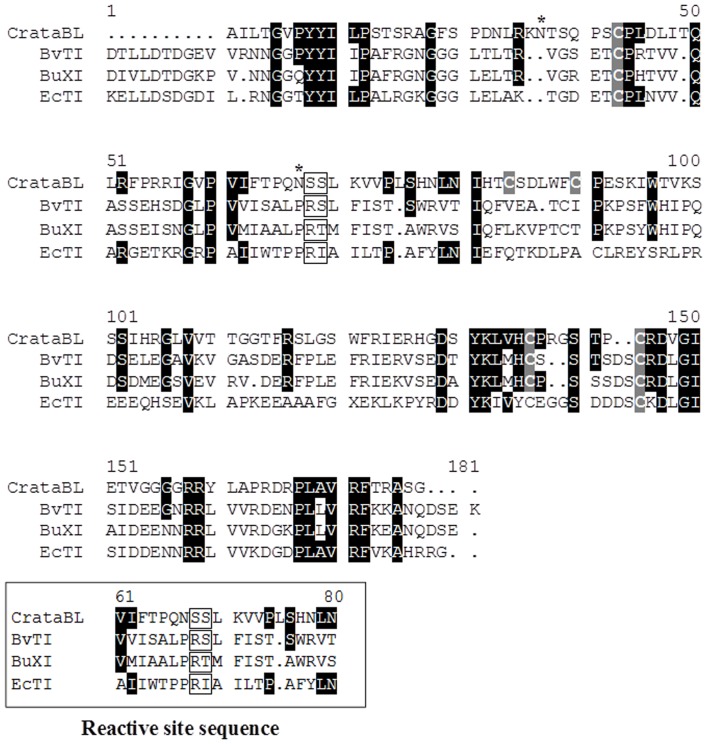
Similarity of CrataBL to protease inhibitors. A comparison of the amino acid sequence of isoform I of CrataBL with the sequences of BvTI – trypsin inhibitor purified from *Bauhinia variegata*; BuXI – inhibitor of factor Xa from *Bauhinia ungulata*; EcTI – trypsin inhibitor purified from *Enterolobium contortisiliquum trypsin inhibitor* [42], [43]. Cysteine residues are shown in gray and highly conserved amino acids are highlighted in black. Residue positions P1 and P1′, between which the proteolytic cleavage occurs, are denoted in boxes. Asterisks indicate glycosylation sites in CrataBL.

The location of the reactive site of CrataBL was determined by chromatography on trypsin-Sepharose in 0.1 M Tris-HCl, pH 8.0. After binding to the support, the inhibitor was kept for 30 min on the column and was then eluted with 0.5 M KCl/HCl pH 2.0 and maintained at this pH. Trypsin bound to Sepharose hydrolyzes specifically the inhibitor at the reactive site and the sample was kept in conditions chosen to avoid the re-ligation of the peptide bond. The resulting modified protein was sequenced and two N-terminal amino acids were found in the eluate, one of them corresponding to the N-terminal Ala1 of the intact inhibitor, whereas the other one could be identified as Ser59. This result indicates that the latter residue must be present in the P1′ position, as defined by Schechter and Berger [46], thus Ser58 must occupy the P1 position. The most potent trypsin inhibitors usually contain a basic residue (Lys or Arg, such as Arg63 in STI [44], [45]) in the P1 position. Only a few known Kunitz-P inhibitors have Ser at P1 position [47], but it has been found that the presence of serine in P1′ enhances the interactions with trypsin [48]. The observed site of cleavage of CrataBL corresponds to the reactive loop in the majority of known Kunitz-P type inhibitors, such as STI [44] (**Figure 4**).

### Crystal structure of CrataBL

**Figure 5:**
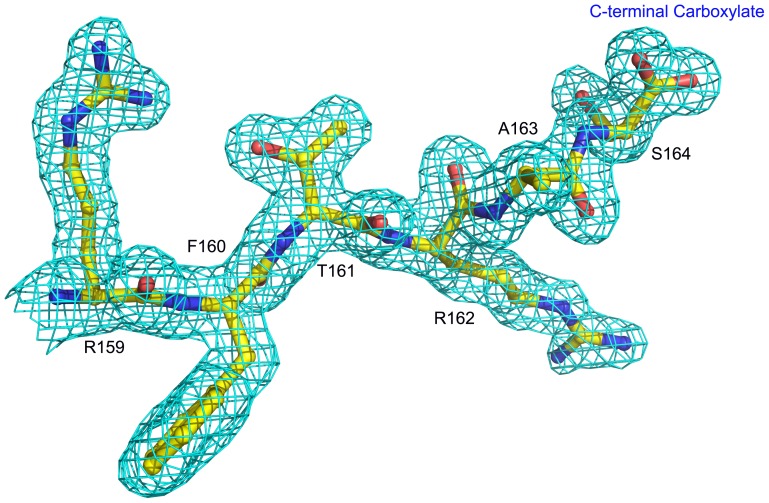
Electron density at the C terminus of molecule A of crystal form I of CrataBL. The 2F_o_-F_c_ map was contoured at 1.2 σ level. The shape of the density corresponding to the C-terminal carboxylate clearly indicates that Gly165, although found in the sequence, is not present in the isoform forming the crystal. Figure prepared with PyMol [69].

The three-dimensional structure of CrataBL was determined in two crystal forms. Both of them were in space group *C*2 and contained two molecules in the asymmetric unit, but the unit cell parameters and crystal packing were different. The structures were refined at comparatively high resolution, 1.5 Å for crystal form I and 1.75 Å for crystal form II, with acceptable geometrical parameters (**Table 1**). The final models were complete for all four independent molecules with residues 1–164 traced. The presence of very well defined C-terminal carboxylate in molecule A of crystal form I suggests that the last residue was Ser164 and that Gly165, although annotated in the primary structure, was not present in the crystallized protein (**Figure 5**). Although, as mentioned above, CrataBL isolated from its natural source is a mixture of isoforms, it appears that primarily molecules of isoform I were crystallizing, since the electron density corresponding to residues that differed between the two isoforms was quite clear. Extensive glycosylation at Asn27 was noted, with up to four carbohydrate units attached to that residue being visible. The best density was present in molecule A of crystal form I, where the typical plant-type glycosylation pattern [Manβ1-4GlcNAcβ1-4(Fucα1-3)GlcNAcβ-Asn] [49] could be observed. The carbohydrates were less well ordered in other molecules of both crystal forms. A weak density that could correspond to a carbohydrate bound to Asn57 in crystal form I did not allow proper modeling. In crystal form II, however, the density adjacent to Asn57 was quite clear and allowed modeling of a covalently bound GlcNAc. Two disulfide bonds were made by Cys33-Cys80 and Cys126-133, whereas Cys74 was present in all molecules in the form of sulfenic acid.

**Figure 6:**
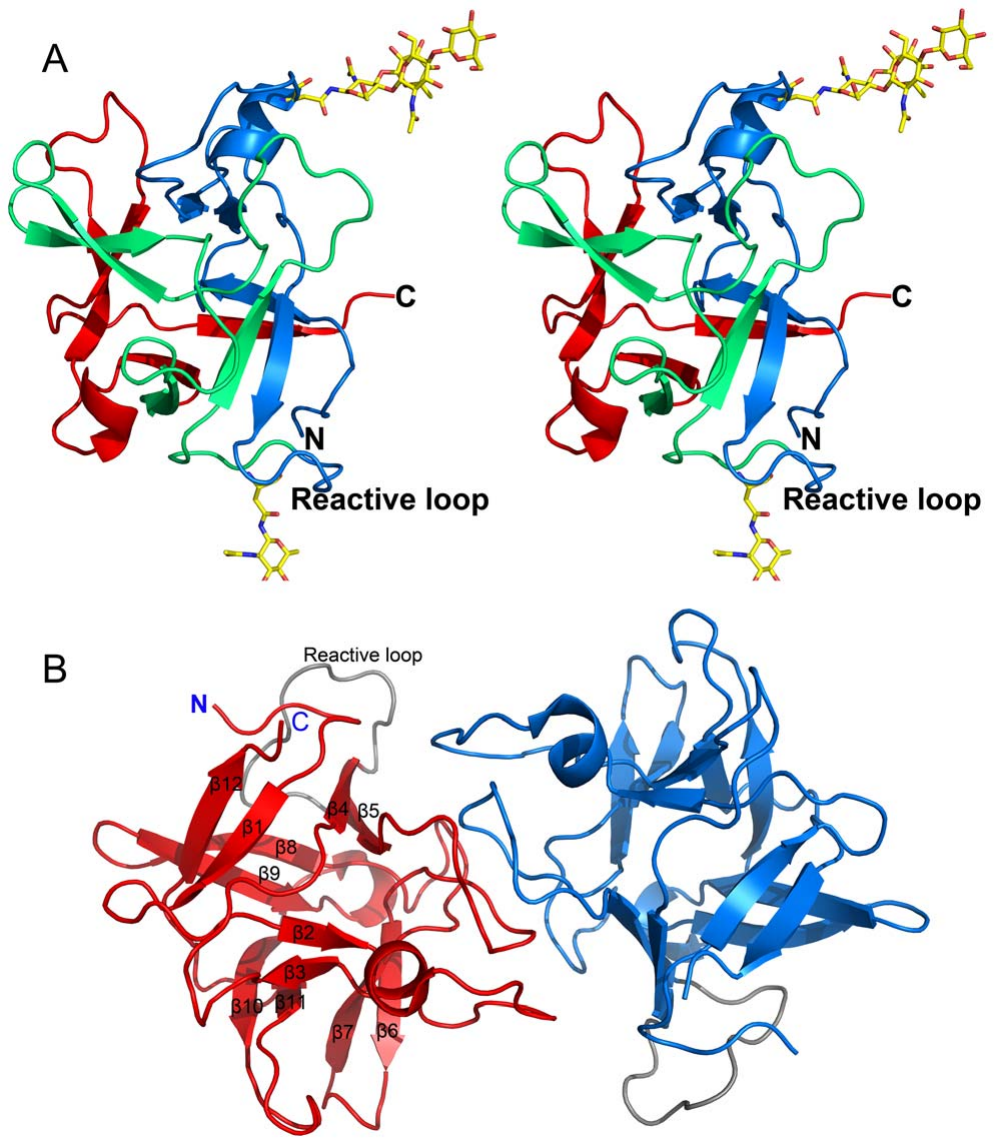
Overall structure of CrataBL. (A) Stereo view of the three-dimensional structure of a CrataBL monomer (molecule A of crystal form I). Three motifs defining the β-trefoil fold are colored in blue, green, and red. N- and C-termini and the putative reactive loop are labeled. Two glycosylated residues and attached carbohydrates are shown in sticks. The carbohydrate attached to the reactive loop is taken from molecule A in crystal form II. (B) A dimer of CrataBL observed in both crystal forms, with the two molecules colored red and blue, respectively. The reactive loops in both monomers are shown in gray. The secondary structure elements are marked on the molecule shown on the left. Figure prepared with PyMol [69].

**Figure 7:**
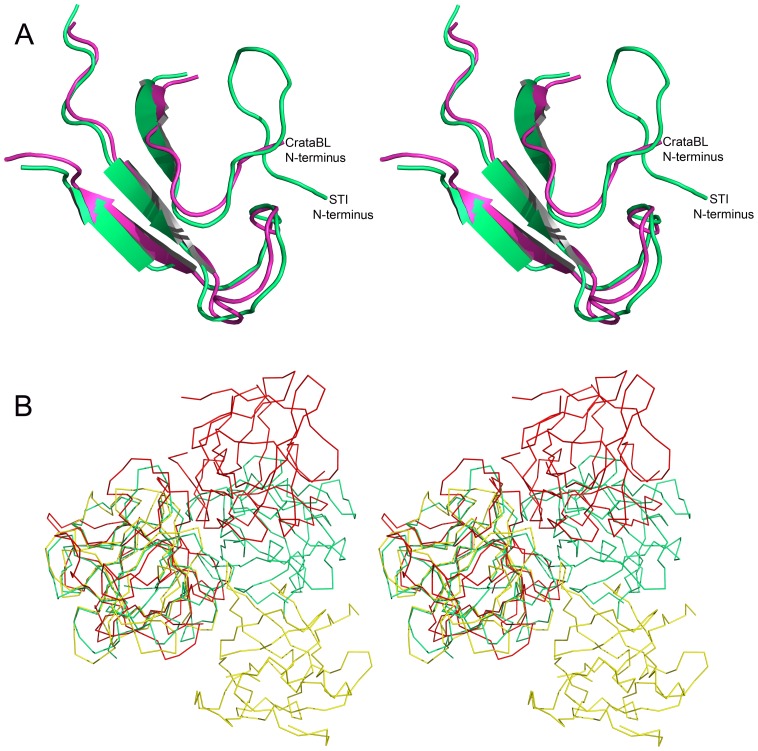
A comparison of CrataBL with proteins sharing the β-trefoil fold. (A) Superimposed regions of the N termini of CrataBL (green) and STI (magenta - PDB ID: 1AVW [44]). (B) A dimer of CrataBL (red) compared to the obligatory dimers of CNL (yellow - PDB ID: 3NBD [9]) and GalNAc/Gal-specific agglutinin from *Sclerotinia sclerotiorum* (green - PDB ID: 2X2S [50]).

The fold of CrataBL consists of a β-trefoil, typical for this protein family. Such a fold contains twelve β-strands arranged into three structurally similar units related by pseudo-threefold symmetry (**Figure 6A,B**). A search of the unique structures in the Protein Data Bank with the DALI server [39] identified at least 14 very closely related proteins, with Z-scores in the range 16.7–19.9 and the reported rmsd of 2.0–2.4 Å for between 140 and 151 Cα coordinates. The alignment of the primary structure of CrataBL with the sequences of these 14 proteins is shown in **Figure 2**. Almost none of the individual amino acids are strictly conserved among all 15 proteins, although pairwise comparisons of their identity with CrataBL are in the 20–30% range. Surprisingly, the protein structurally most similar to CrataBL is the chlorophyll binding protein (PDB ID: 2DRE) which is not a protease inhibitor at all. A number of other proteins that are very similar to CrataBL belong to the miraculin subfamily. Although structural data for miraculin itself are not available, other closely related proteins, for example a miraculin-like protein from *Murraya koenigii* [50], have been studied crystallographically. The clearest sequence and structural difference between CrataBL and the proteins listed in **Figure 2** is its truncation at the N terminus, where about a dozen residues present in all other structures of closely related Kunitz-P inhibitors are not found in CrataBL (**Figure 7A**). These residues form a β-hairpin in the other structures, but their lack does not seem to affect the stability of the fold of CrataBL. On the other hand, a shorter N terminus similar to the one in CrataBL is found in several lectins that exhibit the same overall fold, for exemple in the *Clitocybe nebularis* lectin, CNL [9].

At the concentration of CrataBL used for crystallographic investigations the protein migrates on a column as a dimer (**Figure 6B**), and the asymmetric unit of both crystal forms also contains a pair of molecules. Taking molecule A in crystal form I as a reference, the rms deviation between the Cα from those of molecule B was 0.154 Å, whereas the rms deviations from crystal form II coordinates were 0.197 and 0.221 Å for molecules A and B, respectively. The dimers present in both crystal forms are very similar, with the rms deviation of 0.48 Å for all Cα atoms. The two molecules forming a dimer interact quite extensively with each other, with the contact area per molecule ∼645 Å^2^. The putative reactive loops are not involved in any intermolecular contacts, either within a dimer or with symmetry-related molecules.

However, it is not clear whether the dimer observed in both crystal forms of CrataBL is biologically significant. There is no particular need for dimerization of a protease inhibitor, since Kunitz-P type inhibitors form 1∶1 complexes with their target enzymes. Dimerization was reported to be important for the function of some lectins that belong to the β-trefoil family [9], [51], but the interactions responsible for dimerization of these lectins are very different from those found in the dimer of CrataBL (**Figure 7B**).

Since we were not able to obtain crystals of CrataBL with a target protease such as trypsin, the location of the reactive loop may only be predicted. However, the observation that CrataBL is slowly cleaved by trypsin between serines 58 and 59, located on a loop that is involved in direct interaction with the active site residues in trypsin and other serine proteases [9], lends credence to this assumption. It has been previously shown that Kunitz-P inhibitors are slow substrates and that the cleavage (and re-ligation) site is always located on the reactive loop. This loop is most often located between β strands 4 and 5 (although some other loops may play this role, for example in the potato double-headed inhibitor [52]). Thus the detailed structural basis of the inhibition mode by CrataBL still needs to be verified.

The presence of a verified glycosylation site (Asn57 – **Figure 6A**) on the putative reactive loop might be one of the reasons for the low inhibitory activity of CrataBL. However, such a site is not unique to only this inhibitor. For example, a similarly located site has been also postulated to be present in the miraculin-like protein from *Murraya koenigii* [50], although the published coordinates do not include a carbohydrate moiety attached to Asn64 (the presence of extra density next to that residue is, however, noted). Similarly to CrataBL, the miraculin-like protein is also a comparatively weak trypsin inhibitor.

### Cell viability

**Figure 8:**
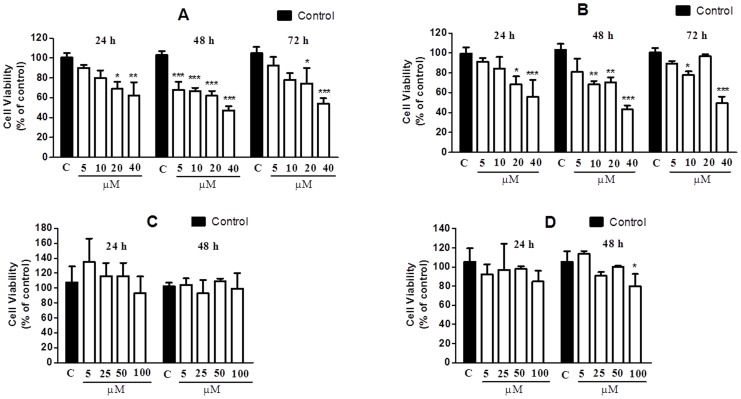
Effects of CrataBL and SbTI on the viability of the DU145 (A, C) and PC3 (B, D) cell lines, respectively. Cells were incubated in 96-well microplates in the concentration of 5×10^3^ cells/100 µL/well. After 24 h, CrataBL, in the final concentrations of 5, 10, 20 and 40 µM, was incubated at 37°C in an atmosphere of 5% (v/v) CO_2_ for 24, 48 and 72 h. The viability was determined by MTT colorimetric method. Results represent the mean ± standard deviation of three experiments, each conducted in triplicate (*p<0.05; ** *p*<0.01; *** *p*<0.001 compared with control cells). In the absence of CrataBL, the 3-(4,5-dimethylthiazol-2-yl)-2,5-diphenyl tetrazolium bromide (MTT) reduction was considered as 100%.

The viability of the DU145 and PC3 cells lines after incubation with CrataBL for 24, 48, and 72 h was investigated by colorimetric MTT assay. Several recent studies have used the MTT assay to determine cell proliferation [53], [54], [55]. The method consists in reducing the tetrazolium salt (MTT) to formazan. As shown in **Figure** 8 (A–B), CrataBL inhibits the viability of the prostate cancer cell lines DU145 and PC3 in contrast to SbTI that did not interfere on DU145 and PC3 cell viability for 24 h (Figure 8 C) and poorly on PC3 for 48 h (Figure 8 D). In the period of 24 h, the cell viability was inhibited in a dose-dependent manner, in response to increasing concentrations of CrataBL (5–40 µM). CrataBL, applied in the concentration of 40 µM for 48 h, inhibited 52.7% and 56.9% of the viability of DU145 and PC3, respectively. In the period of 72 h, the percentage of inhibition of cell viability is lowered. Lectins have been known to bind to cancer cell membranes or receptors causing cytotoxicity, apoptosis, and inhibition of tumor growth [56]. Thus, these results are particularly interesting since they suggest that it might be worthwhile to investigate the effect of CrataBL on prostate cancer cell lines as a possible biotechnological tool. The reduction of MTT is generally attributed to mitochondrial activity, but it has also been related by non-mitochondrial enzymes of the endosomes and lysosomes [57].

### Cell death induced by CrataBL in DU145 and PC3 cell lines

**Figure 9:**
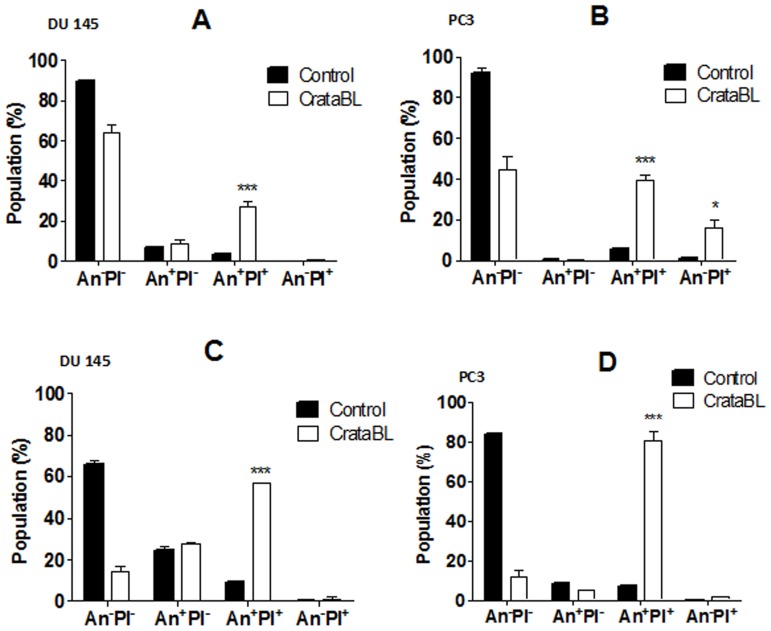
CrataBL induced apoptosis in prostate cancer cells. The cell lines were seeded in 6-well plates for 24 h, followed by washing three times and incubating with RPMI without FBS for cell cycle synchronization. Afterwards cells were treated with CrataBL (40 µM) or medium (control) for 24 h (A and B) and 48 h (C and D). The analysis was performed in FACSCalibur flow cytometer using annexin V-FITC and propidium iodide (PI) staining. The percentage of viable (An^−^PI^−^), apoptotic (An^+^PI^−^), secondary apoptotic (An^+^PI^+^) and necrotic (An^−^PI^+^) cells are represented. Data expressed as mean ± SD, obtained from experiments performed in triplicate. (*p<0.05; *** *p*<0.001 compared with control cells).

**Figure 10:**
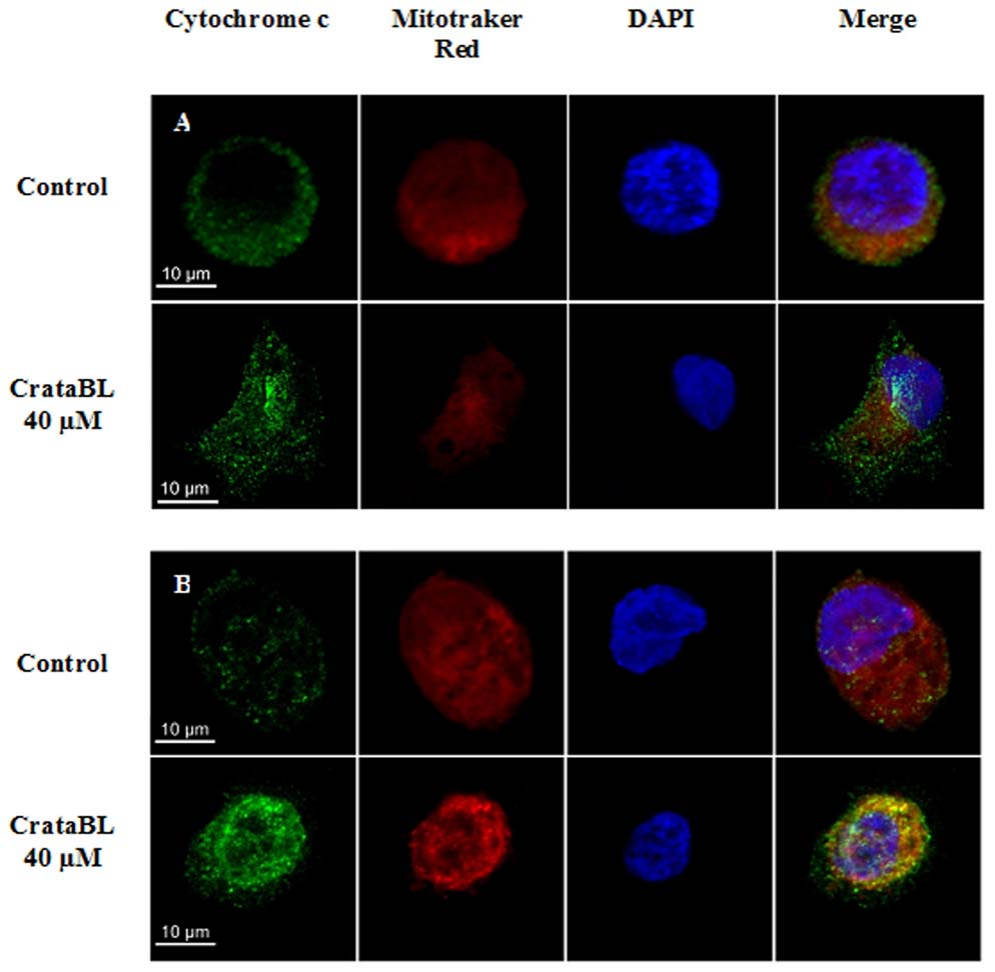
Scanning confocal microscopy of DU145 (A) and PC3 (B) cells (5×10^4^/well). The cells were seeded on 13-mm glass coverslips, placed in 24-well plates and incubated for 24 h at 37°C, in an atmosphere of 5% (v/v) CO_2_. At the end of the incubation period, the cells were incubated with RPMI without FBS for 24 h. Subsequently the cells were treated with 40 µM of CrataBL (12 h) in RPMI without FBS at 37°C, in an atmosphere of 5% (v/v) CO_2_. The cells were washed with PBS and incubated with Mitotracker Deep Red 633 in the dark. The cell lines were marked with a primary antibody mouse anti-cytochrome c, following the incubation with a secondary antibody mouse anti-IgG conjugated with Alexa Fluor 488 (green). The nuclei were marked with DAPI (blue) and the coverslips fixed to slides with fluoromount G. The images were acquired using the confocal scanning microscope Carl Zeiss LSM780. Bar = 10 µm.

**Figure 11:**
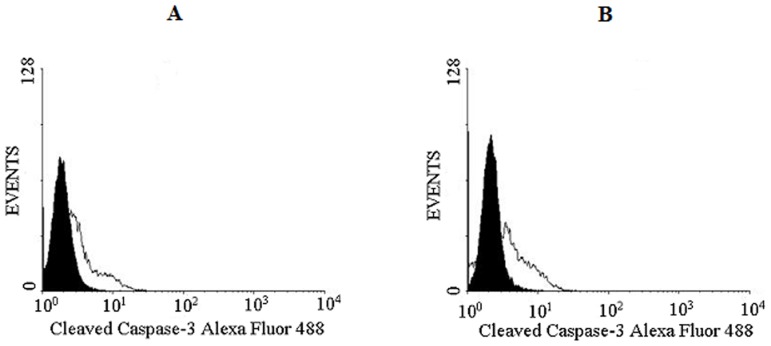
Analysis of caspase-3 activation in prostate cancer cell lines. DU145 (**A**) and PC3 (**B**) (1×10^5^ cells) cell lines were seeded in 6-well plates, following the same protocol for apoptosis with annexin V/FITC and PI staining. Cells treated with CrataBL (40 µM), containing RPMI without FBS were incubated for 48 h at 37°C and 5% (v/v) CO_2_. The cells were incubated with 10 µL of cleaved caspase 3 Alexa Fluor 488-conjugated antibody for 40 min and analyzed in FACSCalibur flow cytometer. As control, the cells were treated with medium only. The area in black represents the control and in white, cells treated with CrataBL.

**Figure 12:**
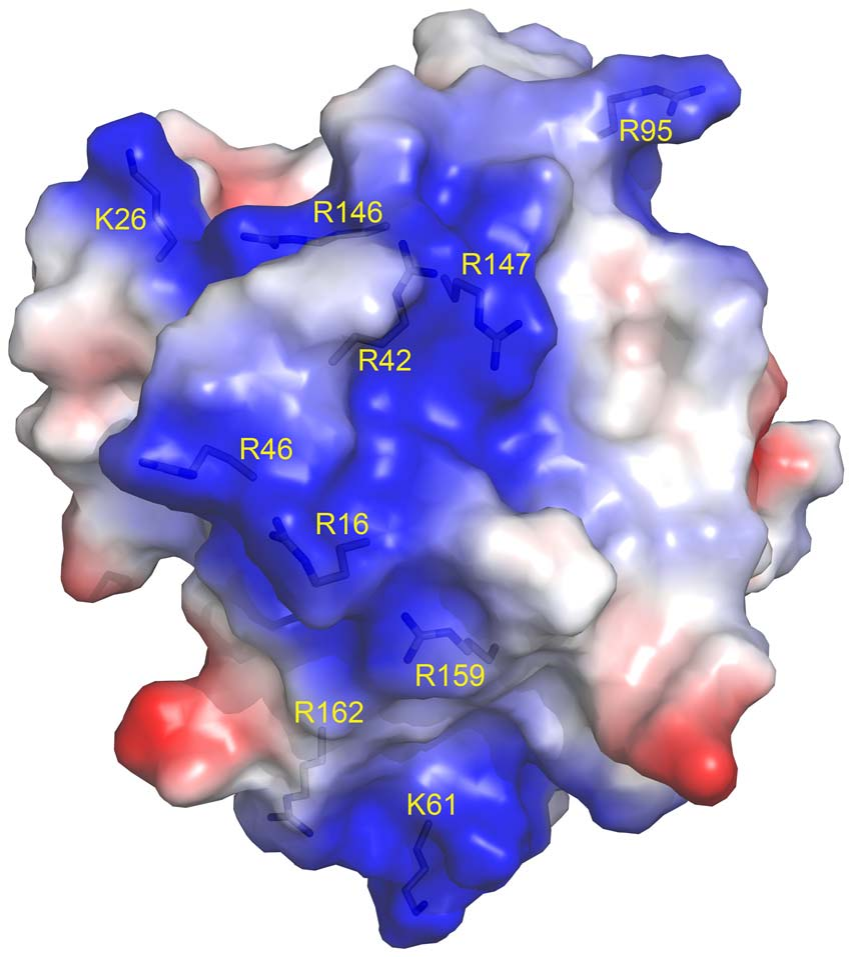
Semi-transparent space-filling representation of the surface of CrataBL molecule colored by residue charges (red negative, blue positive). Arginine and lysine residues which create a contiguous, positively charged channel which may be utilized for binding of sulfated oligosaccharides [64] are labeled. Figure prepared with PyMol [69].

To quantify the manner of cell death, 40 µM of CrataBL was applied to DU145 and PC3 cells for 24 and 48 h, using annexin V/FITC and PI staining. As shown in **Figure 9**, both cell lines exhibit significant death due to apoptosis (*p*<0.001). DU145 treated with CrataBL increased the number of apoptotic cells to 57.1% of the total cell population after 48 h. PC3 cells increased the amount of apoptosis observed in the presence of CrataBL to 80.7%, whereas the apoptotic index for the PC3 control cells was only 8.2%. As apoptosis is also characterized by the loss of mitochondrial function [58] and one of the mechanisms of cell death by apoptosis is the release of cytochrome c by mitochondria [59], an assay to verify such release was carried out (**Figure 10**). CrataBL induces the release of cytochrome c in both cell lines, confirming the induction of cell death in these lines via mitochondria. Apoptosis via mitochondria, also called an intrinsic pathway, is regulated by proteins from the Bcl-2 family which control the permeability of the mitochondrial membrane [60]. The enzymes that are central component of the apoptotic machinery are caspases, cysteine-dependent, aspartate-directed proteases [61]. These enzymes cleave important intracellular proteins associated with apoptosis [62]. CrataBL activates caspase-3 in DU145 and PC3 cells (**Figure 11**), similarly to what Jow et al. [63] found on leukemia cells treated with beauvericin, a cyclic hexadepsipeptide. Zhang et al. [64] showed that CrataBL strongly binds a number of sulfated carbohydrates whereas binding of other carbohydrates is insignificant, concluding that charge-charge interactions are responsible for lectin properties of this protein. In **Figure 12** we show the positively charged surface regions in CrataBL, confirming their basic character. On the basis of these findings we may suggest that CrataBL binds to the surface of the target cells through binding of sulfated glycosaminoglycans, such as chondroitin sulfate and dermatan sulfate. The presence of increased amounts of these carbohydrates in prostate cancer cells has been shown before [65]. Glycosaminoglycans are able to potentiate proteases activities, mainly cathepsins [66], [67] and kallikrein [68]. Thus CrataBL attached to sulfated glycosaminoglycans may interfere with the activities of extracellular proteases, either by directly inhibiting their activity or, indirectly, by shielding the enzymes from the cell surface-bound glycosaminoglycans, impairing the potential of these compounds to potentiate the activity of proteases.

## Conclusions

To conclude, the sequence and three-dimensional structure of CrataBL show extensive similarity to protease inhibitors of the Kunitz-type, although the inhibitory activity is weak (in the micromolar range). Our results suggested that CrataBL activated the intrinsic pathway by caspase-3 activation through of the release of cytochrome c by the mitochondria. The precise mechanism of CrataBL action needs to be clarified, but due to its properties it is possible to postulate that as a lectin it may block the binding of glycoproteins on tumor cell surface, whereas in its inhibitory capacity it may reduce trypsin-like proteolytic activity, thus preventing the processes of malignant invasion and the altered growth control of tumor cells. The importance of the latter activity may be less than of the former one, due to comparatively weak inhibitory activity of CrataBL. However, further investigations are required in order to evaluate the effect of this protein *in vivo* and the structural basis of its activity.
